# γ-Tocotrienol–Loaded Liposomes for Radioprotection from Hematopoietic Side Effects Caused by Radiotherapeutic Drugs

**DOI:** 10.2967/jnumed.120.244681

**Published:** 2021-04

**Authors:** Sang-gyu Lee, Teja Muralidhar Kalidindi, Hanzhi Lou, Kishore Gangangari, Blesida Punzalan, Ariana Bitton, Casey J. Lee, Hebert A. Vargas, Soobin Park, Lisa Bodei, Michael G. Kharas, Vijay K. Singh, Naga Vara Kishore Pillarsetty, Steven M. Larson

**Affiliations:** 1 Department of Radiology, Memorial Sloan Kettering Cancer Center, New York, New York; 2 Molecular Pharmacology Program, Memorial Sloan Kettering Cancer Center, New York, New York; 3 Department of Chemistry, Hunter College, City University of New York, New York, New York; 4 New York University, New York, New York; 5 Hunter College, New York, New York; 6 Department of Pharmacology and Molecular Therapeutics, F. Edward Hébert School of Medicine, Uniformed Services University of the Health Sciences, Bethesda, Maryland; 7 Armed Forces Radiobiology Research Institute, Uniformed Services University of the Health Sciences, Bethesda, Maryland; and; 8 Department of Radiology, Weill Cornell Medical College, New York, New York

**Keywords:** bone marrow, liposome, γ-tocotrienol, radiation protection

## Abstract

With the successful development and increased use of targeted radionuclide therapy for treating cancer comes the increased risk of radiation injury to bone marrow—both direct suppression and stochastic effects, leading to neoplasia. Herein, we report a novel radioprotector drug, a liposomal formulation of γ-tocotrienol (GT3), or GT3-Nano for short, to mitigate bone marrow radiation damage during targeted radionuclide therapy. **Methods:** GT3 was loaded into liposomes using passive loading. ^64^Cu-GT3-Nano and ^3^H-GT3-Nano were synthesized to study the in vivo biodistribution profile of the liposome and GT3 individually. The radioprotection efficacy of GT3-Nano was assessed after acute ^137^Cs whole-body irradiation at a sublethal (4 Gy), a lethal (9 Gy), or a single high-dose administration of ^153^Sm-ethylenediamine-*N,N,N′,N′*-tetrakis(methylene phosphonic acid) (EDTMP). Flow cytometry and fluorescence microscopy were used to analyze hematopoietic cell population dynamics and the cellular site of GT3-Nano localization in the spleen and bone marrow, respectively. **Results:** Bone marrow uptake and retention (percentage injected dose per gram of tissue) at 24 h was 6.98 ± 2.34 for ^64^Cu-GT3-Nano and 7.44 ± 2.52 for ^3^H-GT3-Nano. GT3-Nano administered 24 h before or after 4 Gy of total-body irradiation (TBI) promoted rapid and complete hematopoietic recovery, whereas recovery of controls stalled at 60%. GT3-Nano demonstrated dose-dependent radioprotection, achieving 90% survival at 50 mg/kg against lethal 9-Gy TBI. Flow cytometry of the bone marrow indicated that progenitor bone marrow cells MPP2 and CMP were upregulated in GT3-Nano–treated mice. Immunohistochemistry showed that GT3-Nano accumulates in CD105-positive sinusoid epithelial cells. **Conclusion:** GT3-Nano is highly effective in mitigating the marrow-suppressive effects of sublethal and lethal TBI in mice. GT3-Nano can facilitate rapid recovery of hematopoietic components in mice treated with the endoradiotherapeutic agent ^153^Sm-EDTMP.

## Visual Abstract

**Figure:**
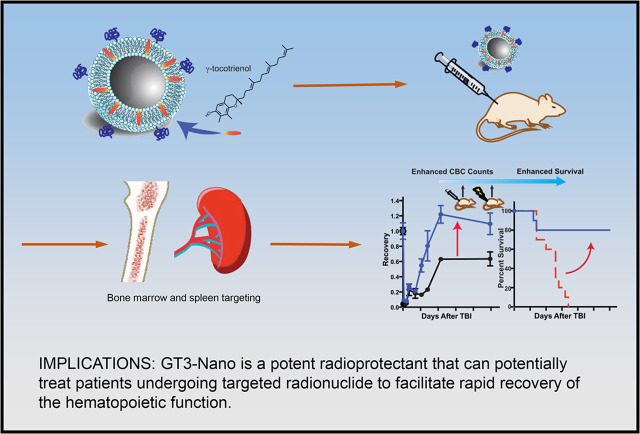

Therapy with ^131^I for well-differentiated thyroid cancer has played a seminal role in the creation of nuclear medicine as a medical subspecialty and, for decades, was the only high-dose targeted radionuclide therapy (TRT) regimen considered the standard of care in oncology. In the last 10 years, prospects for therapeutic nuclear medicine have been further enhanced by the addition of 2 successful new therapeutic radionuclides—for prostate cancer, Xofigo (^223^Ra-chloride; Bayer), and for neuroendocrine tumors, Lutathera (^177^Lu-DOTATATE; Advanced Accelerator Applications)—Food and Drug Administration–approved in 2013 and 2018, respectively. Key registry trials documented improved progression-free survival, and these new therapeutic radiopharmaceuticals are now part of the standard pharmacopeia for advanced adult tumors.

The powerful combination of clinical success, commercial viability, and an opportunity to satisfy a major unmet need for effective therapy of advanced solid tumors in adults and children now provides a powerful stimulus for the development of novel TRTs to encourage further growth of the therapeutic arm of nuclear medicine. Examples of additional radionuclide-targeted therapies showing great promise for treatment of otherwise incurable tumors and in advanced clinical development include ^177^Lu-DKFZ-PSMA-617 for metastatic castration-resistant prostate cancer (in phase 3 testing through the multicenter VISION trial (1)), ^131^I-NaI reinduction therapy for radioiodine-refractory thyroid cancer (2), and ^131^I-omburtamab for central nervous system metastatic neuroblastoma in pediatric patients (3).

As the use of promising TRT agents expands, whole-body radiation doses will also increase, as will the probability of potentially dangerous side effects. These TRTs cause some degree of bone marrow suppression; this organ usually limits tumor dose escalation. For example, reports on ^177^Lu-DOTATATE indicate that mild to moderate hematopoietic complications may be expected in most patients, whereas severe grade 3 or 4 cytopenia occurs in a small but significant (11%) patient population and appears to be correlated with bone marrow dose (4,5). Myeloproliferative events, such as myelodysplastic syndrome and acute leukemia, have been reported in 2.35% and 1.1% of patients in a 30-mo median follow-up (range, 1–180 mo) (6). Similar considerations are postulated for other types of radionuclide therapies (7).

Radioprotectants (7) based on free-radical scavenging capability have been developed over the past few decades to counteract the dangerous side effects of radiation. The radioprotector γ-tocotrienol (GT3) is a vitamin E analog that has demonstrated highly potent radioprotection in mice and nonhuman primates (8). GT3 exerts its radioprotective effects by modulating multiple pathways, including inhibition of hydroxyl-methyl-glutaryl-coenzyme A reductase, induction of expression of the DNA repair gene RAD50, upregulation of thrombomodulin production, and progenitor cell mobilization, which facilitates hematopoietic recovery. However, because of hydrophobicity, GT3 is water-insoluble and has poor bioavailability, making it unsuitable for human applications (9).

We have developed a liposomal formulation of γ-tocotrienol (GT3), or GT3-Nano for short, a radioprotector agent to mitigate hematopoietic effects after TRT and other forms of whole-body radiation. We developed a liposomal carrier for GT3 as a suitable drug formulation for practical solubilization and parenteral delivery of high-dose GT3, as required for optimal radioprotection. In prior work, by adjusting the size, ζ-potential, and polyethylene glycol content, we discovered radiolabeled spleen and bone marrow–targeted liposomal formulations (SBMT-LIPO) that selectively target these areas with enhanced efficiency (10). For the purpose of radioprotection and radiotoxicity mitigation, we reasoned that SBMT-LIPO would be an ideal delivery vehicle because spleen and bone marrow are the primary sites of regenerative stem cells for hematopoietic recovery after radiation. We hypothesized that a water-soluble formulation capable of delivering GT3 to the spleen and bone marrow would demonstrate enhanced radiation protection. In the current article, we describe the synthesis, physiochemical characterization, and in vivo pharmacokinetics of GT3-Nano. The radioprotection properties of GT3-Nano against sublethal and lethal total-body irradiation (TBI), as well as high-dose internal radiation with bone-targeting ^153^Sm-ethylenediamine-*N,N,N′,N′*-tetrakis(methylene phosphonic acid) (EDTMP), were evaluated in immunocompetent mouse models and are presented here.

## MATERIALS AND METHODS

All chemicals were used as received without further purification. All lipids and mini extruder were purchased from Avanti Polar Lipids. The p-SCN-Bn-DOTA (2-[4,7,10-tris(carboxymethyl)-6-[(4-isothiocyanatophenyl)methyl]-1,4,7,10-tetrazacyclododec-1-yl]acetic acid) was purchased from Macrocyclics, and ^64^Cu was purchased from Washington University in St. Louis. GT3 was purchased from Chromadex, and tritium-labeled GT3 was synthesized at ViTrax. Mice were purchased from Envigo Laboratories.

### Preparation of Liposomes and Characterization

Liposomes were prepared as previously described (10). Briefly, DSPC (1,2-distearoyl-sn-glycero-3-phosphocholine), cholesterol, succinyl-DPPE (1,2-dipalmitoyl-*sn*-glycero-3-phosphoethanolamine-*N*-[succinyl]), mPEG2000-DSPE (1,2-distearoyl-*sn*-glycero-3-phosphorylethanolamine), and GT3 were mixed in chloroform, evaporated under N_2_ flow, and lyophilized overnight. Lipid film was hydrated in phosphate-buffered saline (PBS) at 65°C for 1 h, and the lipid dispersion was extruded through a 0.1-μm-pore Whatman polycarbonate membrane filter at 65°C. Liposome size distribution and ζ-potential at 25°C in PBS, pH 7.4, were determined by dynamic light scattering using Zetasizer Nano-ZS (Malvern). Further ^64^Cu labeling was performed by adding ^64^Cu-CuCl_2_ to the liposome at pH 5.5 with 0.2 M sodium acetate buffer at 50°C. The reaction was monitored by instant thin-layer chromatography silica gel paper using a 5 mM diethylenetriaminepentaacetic acid solution.

### In Vitro GT3 Release from Liposomes

GT3-Nano liposomes containing different mole% of GT3 spiked with ^3^H-GT3 148 kBq (4 μCi) were placed inside a dialysis cassette with a molecular weight cutoff of 50,000 and dialyzed against PBS. At 0, 4, 20, 28, 44, 52, 68, and 140 h, 100 μL of the liposomes were withdrawn and transferred to a scintillation vial and mixed with 5 mL of Ecoscint A, and the radioactivity was measured using a scintillation counter (Tricarb 2910 TR; PerkinElmer, Inc.).

### Biodistribution of ^64^Cu-Liposomes and ^3^H-Labeled GT3-Containing Liposomes

For biodistribution studies, approximately 5.5 MBq of ^64^Cu-DOTA-Bz-DSPE–labeled liposomes and 0.74 MBq of ^3^H-GT3–incorporated liposomes were administered to C57/BL6 mice (*n* = 5 per group) via tail vein injection. Cohorts of mice were sacrificed at 24 and 48 h after injection. Major organs were collected and placed in preweighed culture tubes or Eppendorf tubes. For ^3^H activity measurement, major organs were collected in preweighed scintillation vials to which 500 μL of Soluene-350 (PerkinElmer) were added and incubated overnight at 37°C. Ecoscint A, 4.5 mL, was added, mixed well, and counted in a scintillation counter.

### In Vivo Efficacy of GT3 Liposomes Against TBI

Six- to 8-wk-old C57/BL6 mice were used for the whole-body irradiation studies. Cohorts of mice were intravenously administered GT3-Nano (10 mg/kg, 6 mol%) either 24 h before or 24 h after whole-body irradiation (4 Gy; ^135^Cs; dose rate, 82 cGy/min). Approximately 100 μL of blood were drawn using retroorbital collection for complete-blood-cell count data at various time points up to 100 d after radiation. For studies performed at the Armed Forces Radiobiology Research Institute, 6- to 8-wk-old male CD2F1 mice were housed in rooms with a 12-h/12-h light/dark cycle. The mouse holding room was maintained at 20°C–26°C with 10–15 air exchange cycles per hour and a relative humidity of 30%–70%. Mice were held in quarantine for 1 wk and exposed to bilateral radiation in a ^60^Co facility at a dose rate of 0.6 Gy/min.

### Flow Cytometry

Bone marrow cells were harvested by crushing the hind leg bones in a mortar with a pestle in 4-(2-hydroxyethyl)-1-piperazineethanesulfonic acid buffered saline plus 2% fetal bovine serum and passing through a 40-μm cell strainer. To measure hematopoietic stem and progenitor cell compartments, the cells were stained with the appropriate cocktail containing antibodies specific for linage markers. The complete list of antibodies and markers is presented in Supplemental Table 1 (supplemental materials are available at http://jnm.snmjournals.org)**.** Bone marrow cells were analyzed by a BD LSR Fortessa flow cytometer.

### Immunohistochemistry

Seven-week-old C57/BL6 mice were injected with 100 μL of bone marrow–targeting liposomes (NBD-SBMT-LIPO) incorporating the fluorophore nitrobenzoxadiazole (NBD). Once euthanized, mouse spleens were collected and embedded in optimal-cutting-temperature compound and then snap-frozen with dry ice. Frozen spleen sections 10 μm thick were collected on slides and washed 3 times with PBS, followed by incubation with Tris-buffered saline plus 1% bovine serum albumin solution for 1 h. Sections were incubated with mouse anti-CD31 IgG and rabbit anti-CD105 IgG overnight at 4°C. After washing of primary antibody with PBS 3 times, the sections were incubated again with either goat antimouse IgG-Alex-647 or IgG-Alexa-594 for 2 h at room temperature. Sections were mounted on slides and observed under fluorescence microscopy.

### Institutional Research Approval

All animal experiments were approved by the Institutional Animal Care and Use Committee of Memorial Sloan Kettering Cancer Center under protocol 86-02-020 and by the Armed Forces Radiobiology Research Institute under protocol P2017-08-009.

## RESULTS

### GT3-Nano Formulation and GT3 Release Kinetics of ^3^H-Labeled GT3-Nano Were Characterized

**FIGURE 1.**
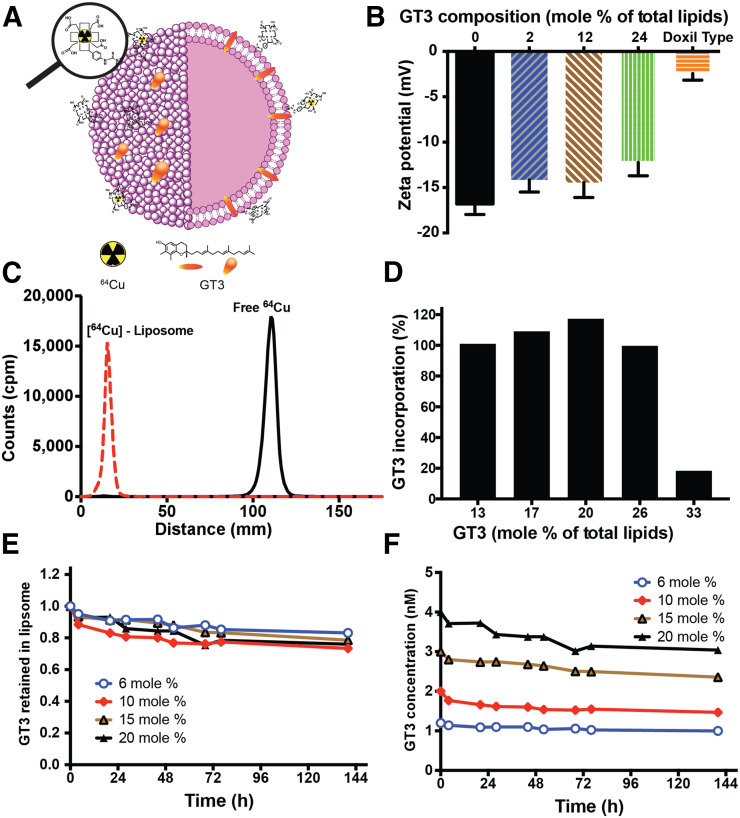
Schematic and characterization of GT3-incorporated bone marrow–targeting liposome. (A) Structure of PET-labeled GT3-Nano. (B) ζ-potentials of SBMT-LIPO. (C) ^64^Cu-GT3-Nano shows 100% labeling of ^64^Cu on instant thin-layer chromatography. (D) GT3 incorporation into liposome. (E and F) In vitro GT3 release kinetics of GT3-Nano shows, at 6, 10, 15, and 20 mol%, GT3 contents in liposome as relatively retained (E) and moles retained (F).

GT3 can be incorporated into bone marrow–targeting liposomes by adding up to 20 mol% GT3 in lipid mixture. The ^64^Cu labeling and stability of the colloidal suspension of GT3-Nano were measured and found to be nearly identical to bone marrow–targeting liposomes (Fig. 1). The maximum loading capacity of GT3 in SBMT-LIPO was determined by adding different amounts of GT3 into SBMT-LIPOs (Fig. 1D); 6, 10, 15, and 20 mol% of GT3 input were tested for liposomal formulation of GT3 for further studies.

Size distribution and negative surface charge play critical roles in high accumulation of SBMT-LIPO in bone marrow (10). Since GT3 incorporation into the lipid bilayer of SBMT-LIPOs modifies the surface property, ζ-potential as well as size distribution have been determined (Fig. 1B). As shown in Figure 1B, ζ-potential of 10 mol% GT3-Nano is −14.4 mV, 2.4 mV higher than the ζ-potential of naïve SBMT-LIPO at pH 7.4. As shown in Figure 1D, GT3 incorporation into SBMT-LIPO does not interfere with labeling of ^64^Cu to DOTA-Bn-DSPE on GT3-Nano, which showed 100% labeling efficiency.

We prepared ^3^H-GT3-Nano containing trace amounts of ^3^H-GT3 as a surrogate for GT3 and measured the release of ^3^H activity from the liposome using dialysis against PBS at different time points up to 140 h at room temperature. In vitro GT3 release was conducted to evaluate the physical stability of GT3 in GT3-Nano, and tritium-labeled GT3 was used to measure release kinetics. As shown in Figures 1E and 1F, the ^3^H-GT3 release rate was measured by dialysis against PBS, and 73%–83% of ^3^H-GT3 was retained in the liposome over 140 h of dialysis at room temperature.

### Biodistribution of ^64^Cu GT3-Nano and ^3^H-Labeled GT3-Nano Showed Similar Accumulation Pattern in Spleen and Bone Marrow

**FIGURE 2.**
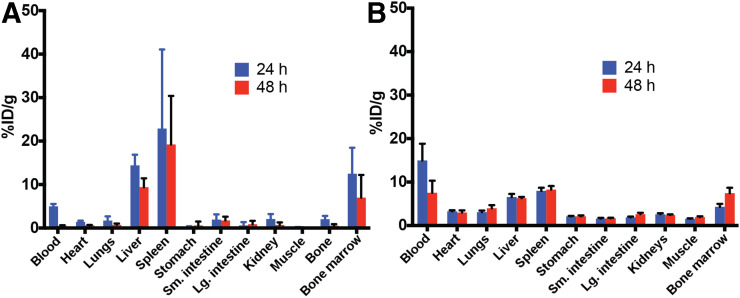
Ex vivo biodistribution data. (A) ^64^Cu-labeled GT3-Nano and (B) ^3^H-GT3-Nano. Ex vivo biodistribution data of ^64^Cu-labeled GT3-Nano and ^3^H-GT3-Nano at 24 h and 48 h, respectively, is presented.

Though the physicochemical changes are minimal, as shown in Figure 1, the biodistribution of GT3-Nano must ensure that the formulation targets the spleen, liver, and bone marrow. We compared SBMT-LIPO, our liposomal radioprotectant, with the spleen and bone marrow. ^64^Cu-labeled and ^3^H-GT3–incorporated GT3-Nano were used to follow the liposomal distribution and GT3 distribution at 24 and 48 h after administration. As shown in Figure 2 and Supplemental Table 2, bone marrow, spleen, and liver accumulations (percentage injected dose per gram of tissue) of GT3-Nano at 24 h were 12.48 ± 2.68, 22.86 ± 8.14, and 14.4 ± 1.1, respectively, as measured by ^64^Cu-GT3-Nano and 4.29 ± 3.22, 7.95 ± 1.61, and 6.53 ± 1.35, respectively, as measured by ^3^H-GT3-Nano, possibly related to differential metabolism of copper and GT3 in phagocytized materials.

### GT3-Nano Mitigated Radiation Effects by Supporting Faster and More Complete Recovery of White Blood Cells (WBCs) and Lymphocytes After Sublethal Whole-Body Irradiation and ^153^Sm-EDTMP Injection

**FIGURE 3.**
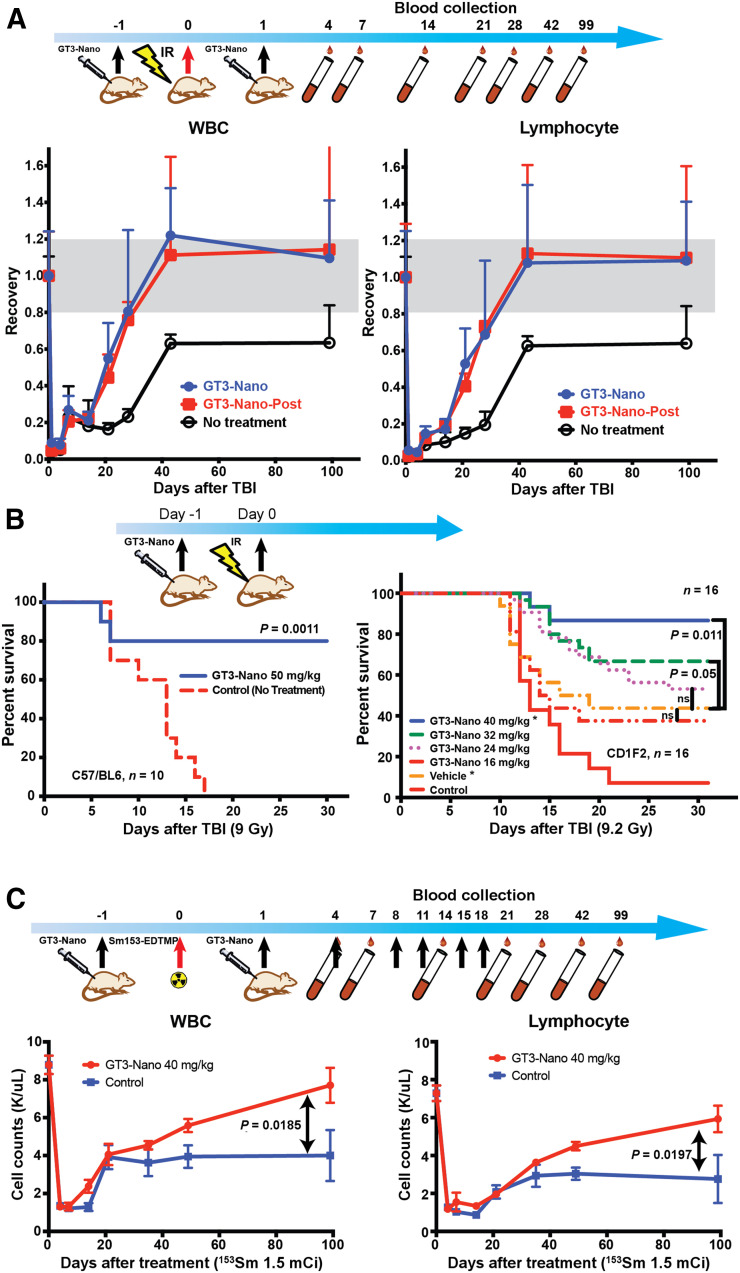
Efficacy of GT3-Nano. (A) WBC recovery after TBI (4 Gy) in 10 mg/kg/mice. (B) Dose-dependent survival improvement in mice at lethal radiation doses. (Left) C57/BL6 mice were administered GT3-Nano. (Right) CD2F1 mice were administered GT3-Nano at various GT3 dosages, and the lipid mass of SBMT-LIPO was the same as the lipid mass of 40 mg/kg GT3-Nano. Data for control group (no treatment) were obtained from Singh et al. (12). (C) WBC recovery of ^153^Sm-EDTMP treatment with GT3-Nano. GT3-Nano-Post = GT3-Nano after irradiation; IR = irradiation; ns = not statistically significant.

As shown in Figure 3A, 4 Gy of whole-body radiation induced severe depletion of peripheral blood cells. WBCs and lymphocytes decreased to less than 10% of baseline counts, with a nadir at days 1–4 after irradiation, consistent with our previous reports (11). The rate of recovery of WBCs and lymphocytes in GT3-Nano–treated groups (both before and after irradiation) was faster, leading to more than 50% recovery by day 21 compared with untreated controls, for which recovery was about 20% by day 21. Both treated groups also demonstrated complete recovery of key parameters to baseline by day 43. For the untreated control group, WBC and lymphocyte counts recovered to about 60% at day 43 and we did not observe 100% recovery until day 99, at which point all the mouse groups were euthanized. GT3-Nano treatment did not affect neutrophil, red blood cell, or platelet counts at 4 Gy of whole-body irradiation (Supplemental Fig. 1).

To evaluate the efficacy of GT3-Nano at a lethal dose of whole-body irradiation, C57/B6 mice (Fig. 3B, left panel) were first tested after irradiation with 9 Gy of ^137^Cs. After GT3-Nano treatment in the amount of 50 mg/kg, the survival rate of the mice was 80% at day 30, in contrast to a 0% survival rate in the untreated group at day 17 at the same irradiation level.

To test the dose dependency of survival with GT3-Nano treatment, CD2F1 mice were treated with 16, 24, 32, and 50 mg/kg of GT3-Nano and SBMT-LIPO (Fig. 3B, right panel). The difference between the survival of mice in the 16 and 24 mg/kg groups and in the group receiving SBMT-LIPO was not statistically significant. When comparing with untreated mice (12), we did observe statistically significant differences between untreated controls and GT3-Nano groups at all doses. The survival of the mice was 67% (*P* < 0.05) in the 32 mg/kg treatment group on day 30 and 87% (*P* < 0.05) in the 50 mg/kg group on the same day. Thus, we confirmed that GT3-Nano can rescue mice of 2 different strains from otherwise lethal radiation in a dose-dependent manner at 2 different institutions.

To test the ability of GT3-Nano to protect bone marrow from internal, exponential declining and continuous radiation after irradiation with a 1.93-d half-life, mice were treated with ^153^Sm-EDTMP, a bone-targeting radiopharmaceutical commonly used as an agent to palliate bone pain caused by bone metastases of cancers. A 50 mg/kg dose of GT3-Nano was administered 24 h before injection of 55.5 MBq (1.5 mCi) of ^153^Sm-EDTMP, followed by six 50 mg/kg injections of GT3-Nano over 3 wk (days 1 and 4 of each week). As shown in Figure 3C, GT3-Nano pretreatment did not prevent the depletion of WBCs and lymphocytes, and as in untreated controls, the WBC and lymphocyte counts dropped to 15% of baseline at day 4. Recovery of WBCs was delayed until day 7 for both the GT3-Nano–treated group and the control group. The nadir region continued to day 14 for the control group, whereas the WBC counts of the GT3-Nano–treated group started to recover at day 14. We found that the recovery of WBCs in the GT3-Nano–treated group was faster and more complete, reaching 88% over 99 d, whereas recovery of WBC counts in the control group reached 44% at day 21 and was still suppressed at the time of sacrifice, day 99 (*P* < 0.05).

### Flowcytometry Showed Faster and More Complete Recovery of the Precursor Hematopoietic Cell Populations in Bone Marrow, After Injection of GT3-Nano

GT3 is known to inhibit hydroxy-methyl-glutaryl-coenzyme A reductase and induce production of granulocyte colony-stimulating factor and expression of DNA repair gene RAD50, as well as other unknown mechanisms (8,9). Although uptake of GT3-Nano occurs in CD105^+^ endothelial cells, it is unclear which cell population is repopulated more rapidly in the bone marrow of GT3-Nano–treated animals.

**FIGURE 4.**
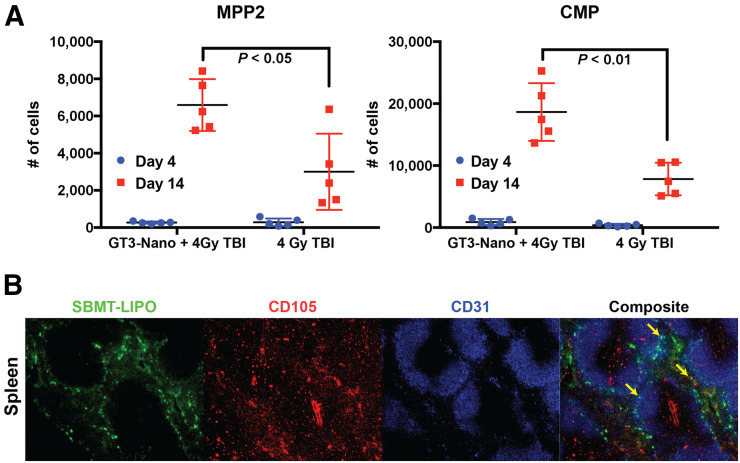
(A) GT3-Nano–treated mice demonstrate improved recovery of HSC cell subpopulation changes in bone marrow, with statistically significant increase in MPP2 and CMP. (B) Immunohistochemistry of C57BL/6 mouse frozen spleen section. Composite imaging shows that SBMT-LIPO is colocalized mostly with CD105. There are a few regions (arrows) where SBMT-LIPO, CD105, and CD31 are colocalized.

Hematopoietic stem cells (HSCs) and multipotent progenitors (13) are responsible for replenishing other blood cells through the hematopoietic process, though HSCs exist mostly in a state of quiescence, or reversible growth arrest. The altered metabolism of quiescent HSCs helps the cells survive for extended periods. When provoked by cell death or damage, HSCs exit quiescence and begin actively dividing again (13). There are several markers that permit the prospective identification and isolation of HSCs and multipotent progenitors (MPPs). HSC and MPP frequency changes in bone marrow were analyzed to identify which HSC/MPP subpopulations are protected by the GT3-Nano treatment. As shown in Fig. 4A), at day 14, myeloid-biased MPP subset 2 (MPP2) and common myeloid progenitors (CMP) showed statistically significant increases in cell numbers during recovery in GT3-Nano–treated groups, in comparison to other HSC subpopulations. Although there was a general trend toward increased progenitor cell subpopulations in the GT3-Nano–treated group, no statistically significant increases were observed in these other hematopoietic stem and progenitor cell populations (Supplemental Fig. 2).

### CD105 Colocalized with Fluorescently Labeled SBMT-LIPO

As shown in Figure 4B, NBD-SBMT-LIPO primarily localized in endothelial cells lining the sinusoids within the red pulp region in the spleen, where sinusoids are engorged with blood and macrophages. CD105^+^ appears red on vascular and sinusoidal endothelial cells, where activated macrophages and monocytes are seen, with NBD-SBMT-LIPO colocalization. CD105^+^ cells are localized mainly in the red pulp region, and some CD105^+^ cells are found in the white pulp region, where lymphocytes are abundant. CD31^+^ has been found on endothelial cells, platelets, macrophages and Kupffer cells, and lymphocytes. CD31^+^ cells in the spleen are located mainly in the marginal zone between the nonlymphoid red pulp and B-cell–abundant white pulp. Although abundant NBD-SBMT-LIPO colocalized with CD105^+^ cells, NBD-SBMT-LIPO also colocalized with CD31^+^/CD105^+^ cells (arrows in Fig. 4B), which are known to produce IL-33 with radiation (14).

## DISCUSSION

Particles such as liposomes, when introduced into the bloodstream, are rapidly cleared from the circulation by endothelial cells of the innate immune system in the liver, spleen, bone marrow, and lymph nodes (15). Liposome can be made of different components of lipids, and when different components of the lipids were used, liposome targets different organs (Supplemental Fig. 3). The phagocytic endothelial cells of spleen and bone marrow line the blood vessels, which have a sinusoidal architecture that has adapted to allow blood cells to rapidly pass back and forth through holes in the vessel walls into adjacent cellular compartments. We reasoned that if we loaded liposomal particles with a known radiation mitigator or protector such as GT3, we could protect these sinusoidal endothelial cells, as well as other essential stem cell types in the spleen and bone marrow that are responsible for restoration of hematopoietic function after radiation damage by TRT radiopharmaceuticals and after other types of acute radiation injury. The physical juxtaposition of the bone marrow niche for HSCs and progenitor cells within 10 μm of the sinusoids in bone marrow and spleen further supports this concept (16).

The studies reported in this paper demonstrate the efficacy of GT3-packaged liposomal formulations for the treatment of radiation-induced hematopoietic injury in mice. We used both acute sublethal and lethal external-beam radiation and high-dose ^153^Sm bone-seeking radiation in mice to test efficacy. The principal benefits of therapy with GT3-Nano were more rapid and complete recovery of WBCs at sublethal radiation (4-Gy external-beam; ^153^Sm overdose), and this recovery correlated with an increase in specific myeloid progenitor cells in the bone marrow. At supralethal radiation (≤9.2 Gy), a dose response was observed for radiomitigation, leading to rescue (survival) of 80%–90% of mice, with no detectable long-term effects after 100 d.

This work was predicated on prior studies that showed a strong link between hematopoietic cells and phagocytic sinusoidal endothelial cells in the bone marrow in both rabbits (17) and humans (18). The 2 cell populations exist naturally in a homeostatic relationship. Radiation disrupts this relationship. At low doses of radiation, the more sensitive hematopoietic cells were markedly suppressed, whereas the phagocytic cells were relatively unaffected. Over time, the homeostatic relationship was restored by the recovery of hematopoietic cells to baseline levels. At higher doses of radiation, both cell types were suppressed, although function of the phagocytic cells declined much more slowly. Recovery of hematopoietic cells was restored only if the function of phagocytic cells was completely restored (6). More modern studies support this concept; Hooper et al. stated, “We show that the extent and rate of [sinusoidal endothelial cell] regeneration after myelosuppression dictate the degree of hematopoietic recovery.” (19).

Thus, protection of the human body from radiation exposure is a major unmet need for TRT, as well as for other human endeavors such as space exploration and military and industrial applications, which are outside the scope of this paper. So far, bone marrow stimulants (e.g., lymphokines such as filgrastim and derivatives) have provided valuable but limited support, and more effective drugs are sorely needed (8). Forms must be stable to long-term storage in strategic national stockpiles and for use as needed with TRT, likely on the shelves of radiopharmacies. Herein, we report practical formulations of GT3-Nano with long-term stability. The carrier liposomes themselves are stable to physiochemical testing for 2 y when stored at 4°C as a kit formulation (Supplemental Figs. 4 and 6). The safety profile, well in excess of effective marrow-sparing doses, appears excellent, with no evident histopathology after doses above 100 mg/kg in mice (Supplemental Fig. 6).

With regard to the mechanism of action, based on fluorescent tags on the liposomal particles, we determined that, as expected, specialized phagocytic cells of the innate immune system lining the sinusoids of bone marrow and spleen are the predominant sites of uptake and retention of these liposomes. GT3-Nano accumulates in these sinusoidal endothelial cells, including CD105^+^ cells and CD31-expressing cells known to produce IL33 lymphokine, a peptide stimulus that activates stem cells and progenitor cells (14). In treated animals, we demonstrated increased MPP2 and CMP progenitor cell populations in bone marrow. In preliminary studies, we have documented that at GT3-Nano doses effective for sublethal irradiation protection (10 mg, GT3/kg), there is no reduction in the efficacy of curative external-beam treatment of human xenografts (Supplemental Fig. 7). Since GT3-Nano shows efficacy in mitigating acute radiation effects, longer-term studies on humans are needed to determine the impact on reduction of stochastic effects.

GT3-Nano affords radioprotection against both single high-dose-rate γ-radiation delivered in 10 min with a ^137^Cs source and slow-dose-rate β-radiation delivered exponentially in about 10 d by ^153^Sm-EDTMP. Both β- and γ-radiation cause most cellular damage by oxidative stress (20), and therefore it is not surprising that GT3-Nano can provide radiation protection against both types of radiation. However, repeated dosing was used for radioprotection by GT3-Nano against ^153^Sm-EDTMP. Additional radiobiologic studies comparing and contrasting radiation protection mechanisms as a function of dose rate and mode of delivery are planned.

## CONCLUSION

We have successfully developed GT3-Nano as a novel water-soluble liposomal drug delivery formulation of GT3, a known ion scavenger drug. GT3-Nano selectively targets the spleen and bone marrow with high efficiency and provides radiation protection for progenitor stem cells against lethal whole-body radiation in mouse models. GT3-Nano promotes more rapid hematopoietic recovery from high-dose radiation both from external sources and from the internal radiation emitter (^153^Sm-EDTMP). We believe that GT3-Nano has major potential for the treatment of nontarget bone marrow toxicities arising from whole-body radiation from external-beam and targeted radiotherapeutics.

## DISCLOSURE

Funding support from P30 CA008748 (NIH/NCI Cancer Center support grant), R01 CA201250-04, P50 CA086438 (Steven Larson), and a MSKCC-IMRAS seed grant (Sang-gyu Lee, Naga Pillarsetty) is gratefully acknowledged. The study at the Armed Forces Radiobiology Research Institute was supported by intramural grant RAB29173 to Vijay Singh. Sang-gyu Lee, Naga Pillarsetty, and Steven Larson are listed as the inventors on the intellectual property related to liposomes for delivering drugs to the spleen and bone marrow. The opinions or assertions contained herein are the private views of the authors and not necessarily those of the Armed Forces Radiobiology Research Institute, the Uniformed Services University of the Health Sciences, or the Department of Defense. No other potential conflict of interest relevant to this article was reported.

**KEY POINTS**

**QUESTION:** With the increasing use of TRT, novel approaches are needed to reduce nontarget organ toxicity.
**PERTINENT FINDINGS:** By incorporating GT3 in SBMT-LIPO, GT3-Nano showed efficacy in increasing blood cells and in rescuing mice from lethal dose of irradiation.
**IMPLICATIONS FOR PATIENT CARE:** GT3-Nano is a potent radioprotectant that can potentially treat patients undergoing TRT to facilitate rapid recovery of the hematopoietic function.
