# Supplementary material for: γ-Tocotrienol–Loaded Liposomes for Radioprotection from Hematopoietic Side Effects Caused by Radiotherapeutic Drugs
Source: J Nucl Med. 2021 Apr;62(4):584–90. doi: 10.2967/jnumed.120.244681 (PMC8049360; doi:10.2967/jnumed.120.244681)
Supplement: Supplementary file 1 [file jnm244681SupplementaryData.pdf]

# Supplementary Data

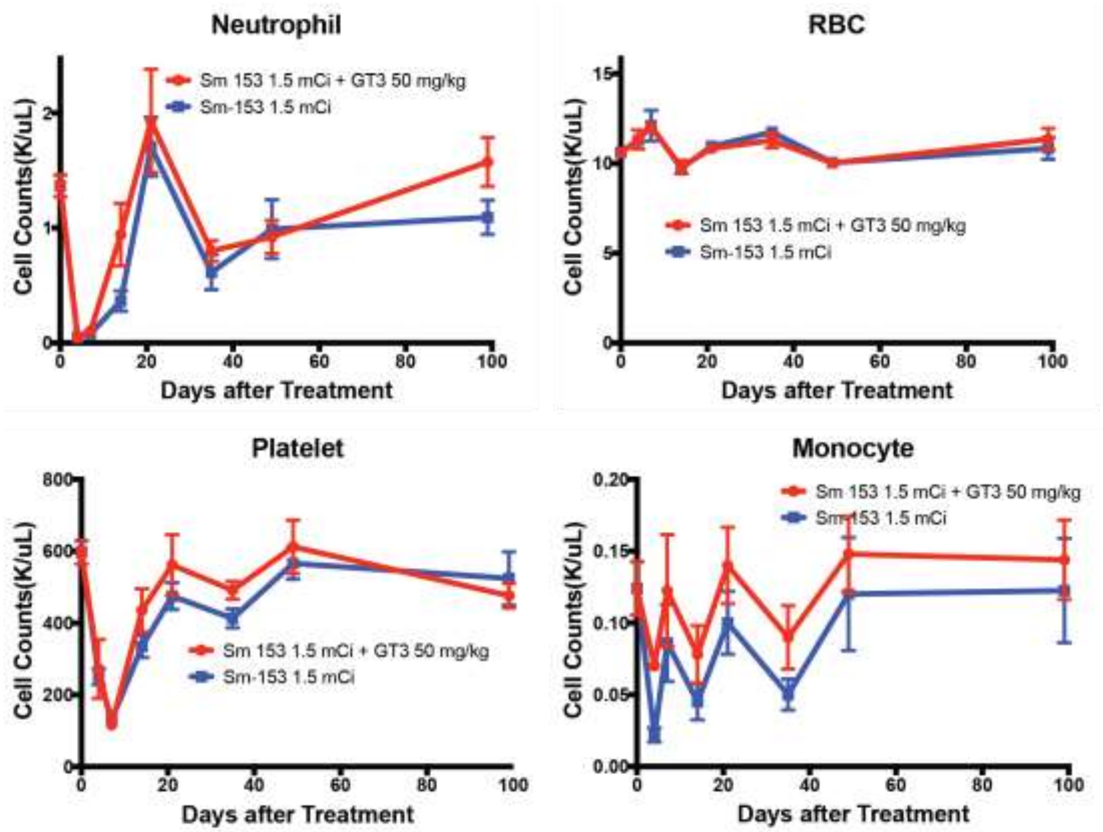


**Supplemental Figure 1. Blood cell recovery of [$^{153}$Sm]-EDTMP treatment with GT3-Nano**. [$^{153}$Sm]-EDTMP and GT3-Nano injection schedule and blood collection scheme: 1.5 mCi of Sm-153 was administered intravenously to two groups (5 mice per group) of mice. 40 mg/kg GT3-Nano was administered 24 h prior to [$^{153}$Sm]-EDTMP injection and an additional 40 mg/kg GT3-Nano was administered on days 1, 4, 8, 11, 15, and 18. Blood was collected on days 4, 7, 14, 21, 28, 42, and 99. The data is presented as mean ± SEM.

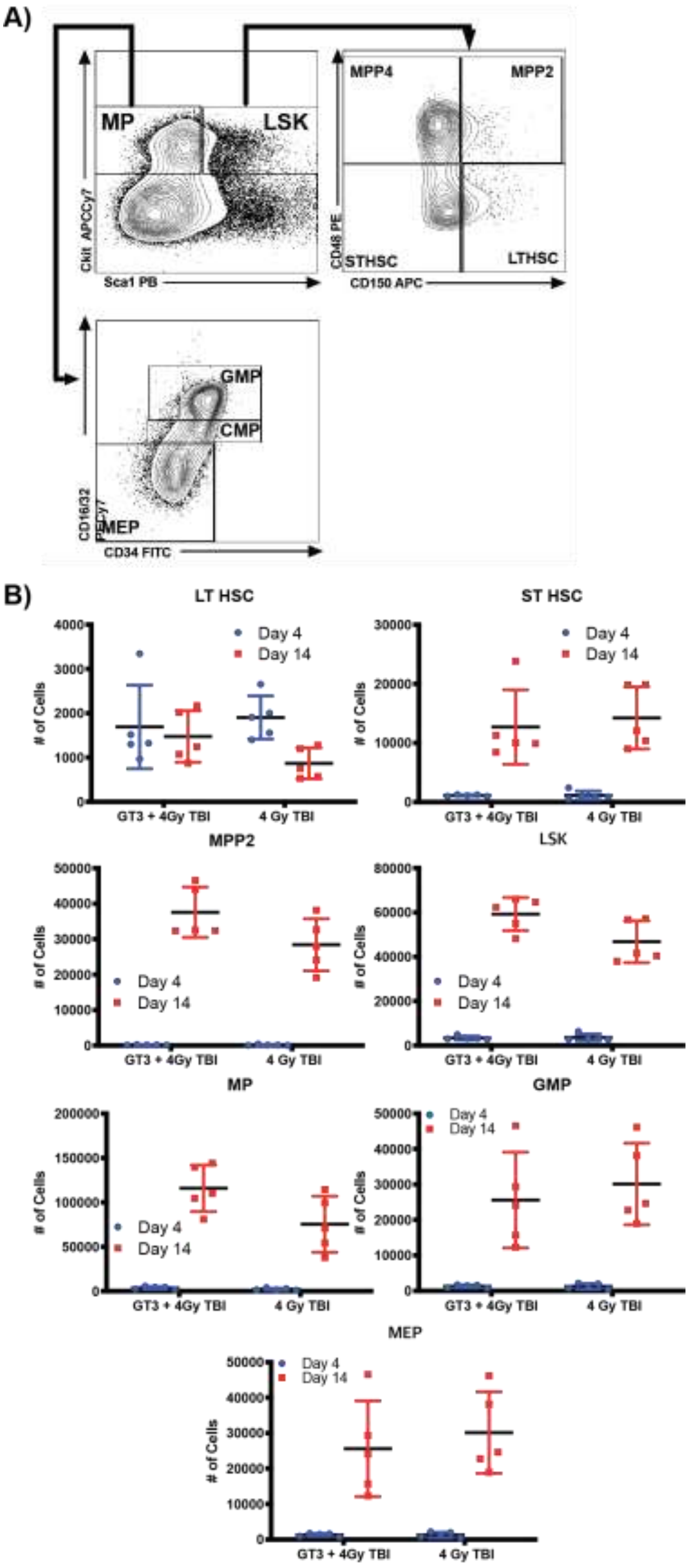
A)
MP
LSK
Ckit APCCy7
Sca1 PB
MPP4
MPP2
CD48 PE
STHSC
LTHSC
CD150 APC
GMP
CMP
MEP
CD16/32 PECy7
CD34 FITC
B)
LT HSC
ST HSC
MPP2
LSK
MP
GMP
MEP
Day 4
Day 14
# of Cells
GT3 + 4Gy TBI
4 Gy TBI

**Supplemental Figure 2. HSC and HSPC cell population changes in bone marrow**. A) gating strategy for HSPC (hematopoietic stem and progenitor cell) populations. B) After C57BL/6 mice were irradiated at 4 Gy with or without GT3-Nano, bone marrow was collected and analyzed by FACS. Myeloid-biased MPP subsets 2 (MPP2) and common myeloid progenitors (CMP) showed statistically significant recovery as compared to other HSPC and progenitor populations.

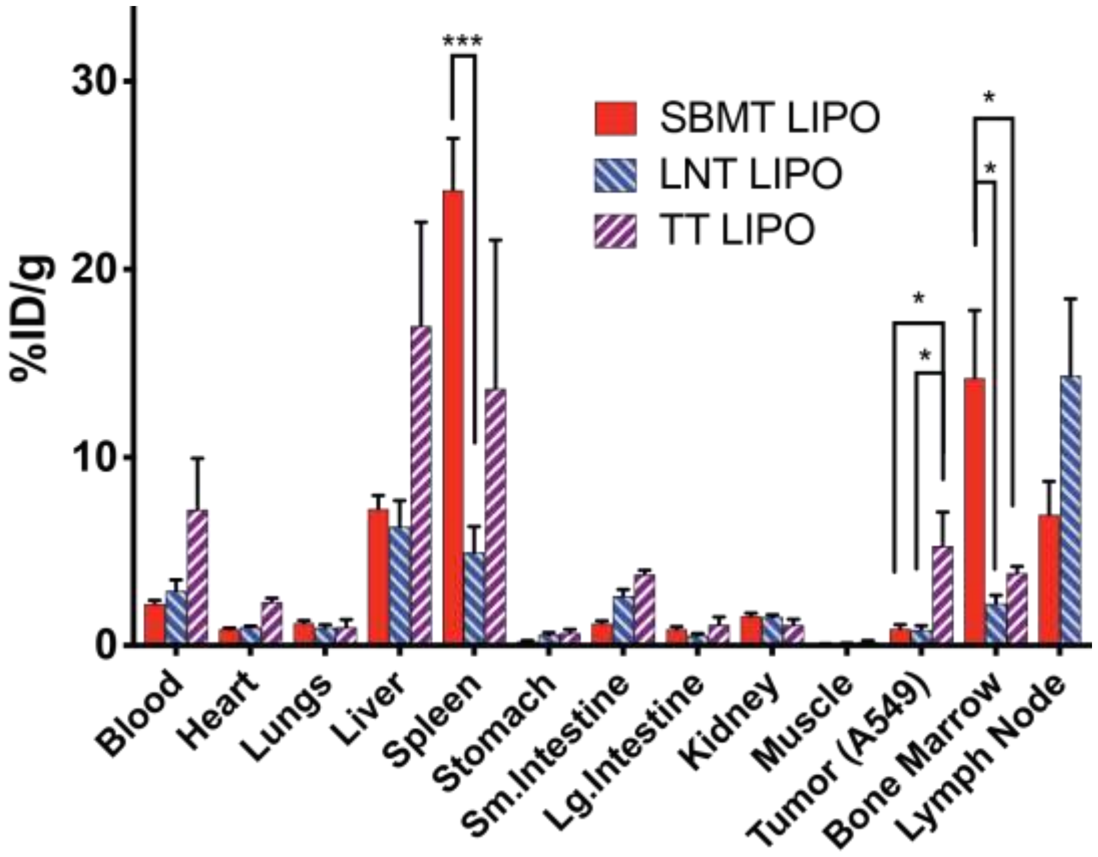


**Supplemental Figure 3. Biodistribution of [$^{64}$Cu]-labeled selective targeting liposomes at 24 h post-injection.** Athymic nude mice bearing A549 tumor were injected with ~140 μCi (5.18 MBq) of $^{64}$Cu-labeled liposomes. 100 mg (2 μmoles) of lipid was injected into each mouse through *i.v.*, corresponding to approximately 6 x $10^{12}$ liposome particles per mouse. Mice were euthanized at 24 h post-injection and organs were harvested in pre-weighed tubes to measure organ weight and γ-counting. N=5 per group. Data was presented as mean and SEM. * $p < 0.05$, *** $p < 0.001$ SBMT: spleen and bone marrow-targeting liposomes; LNT: lymph node-targeting liposome; TT: tumor-targeting liposomes.

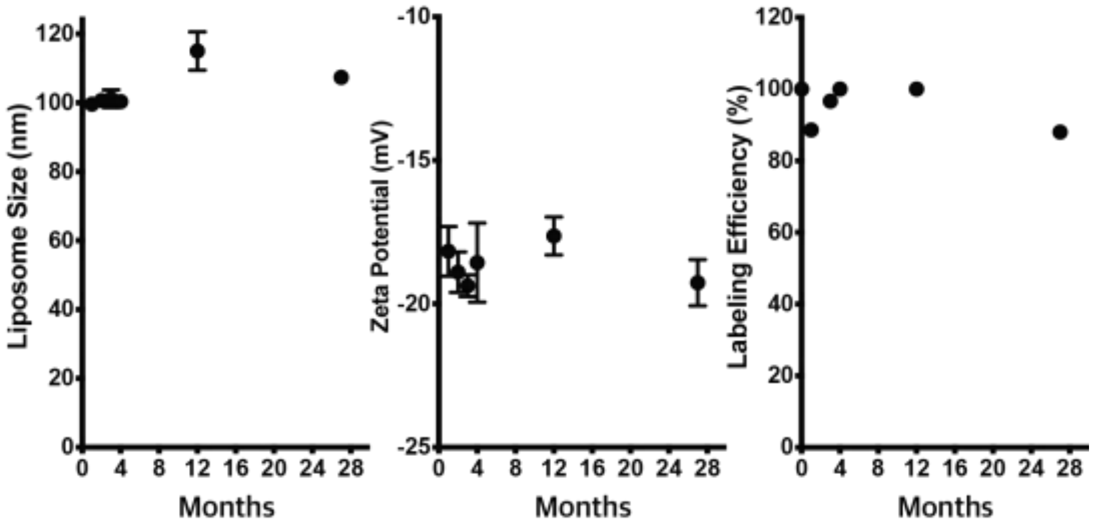


**Supplemental Figure 4. SBMT-LIPO stability.** SBMT-LIPO (20 mM total lipids) was stored at 4 $^{o}C$ and the size, zeta potential, and $^{64}Cu$ labeling efficiency was measured at 1, 2, 4, 6, 12, and 28 months. As seen above, SBMT-LIPO size, zeta potential, and labeling efficiency remain similar for 28 months.

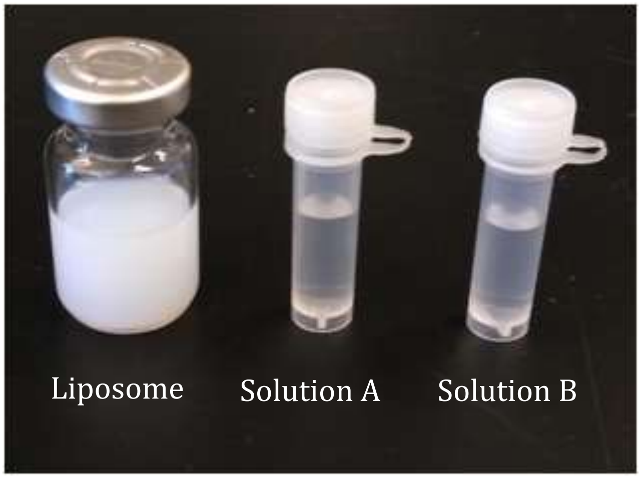


**Supplemental Figure 5. SBMT-LIPO as a "kit" formulation** has been developed to label $^{64}$Cu if needed. Solution A is 200 mM sodium acetate buffer (pH 5.0) and Solution B is 1 M NaOH. [$^{64}$Cu]-BMT-LIPO injection is prepared using the following aseptic procedure: Aseptically transfer 125 uL of solution A to 5 mL SBMT-LIPO vial at 50 $^{o}$C and mix the solution by gentle swirling. Add aseptically desired activity of [$^{64}$Cu]-$CuCl_2$ into SBMT-LIPO vial and mix the solutions by gentle swirling. Place SBMT-LIPO vial in 50 $^{o}$C heating block or heating bath for 30 minutes. Aseptically add 32 uL of neutralizing solution to BMT-LIPO vial. Labeling efficiency will be tested using iTLC with 5 mM DTPA.

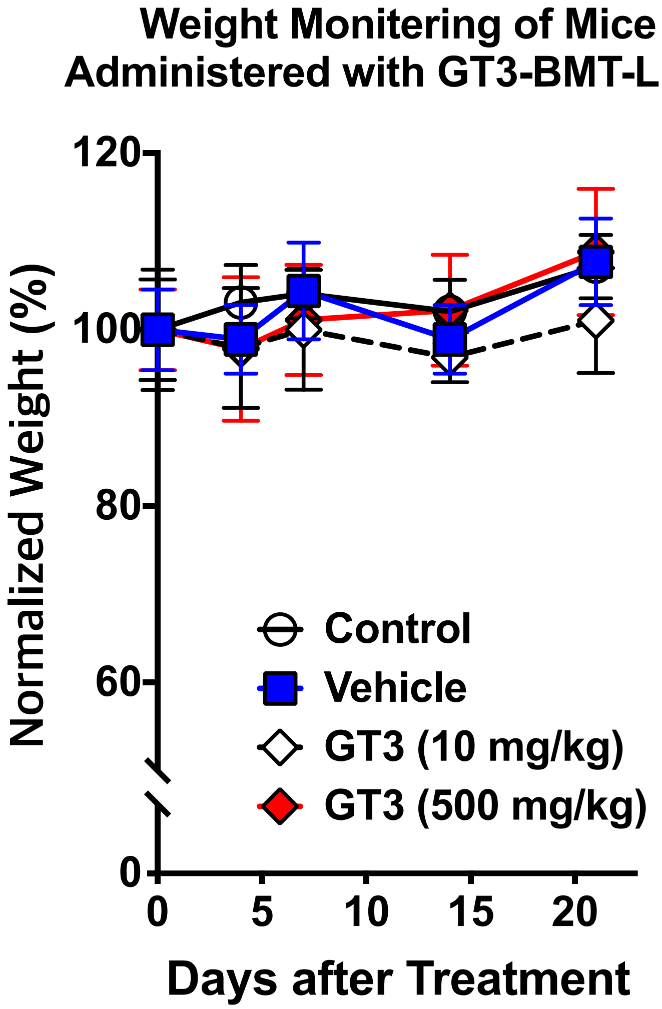


**Supplemental Figure 6. Body weight change with SBMT-LIPO and GT3-Nano**. C57/BL6 mice were administered SBMT-LIPO (2.5 g/kg of total lipid) and GT3-Nano (10 mg/kg GT3 + 50 mg/kg total lipid or 500 mg/kg GT3 + 2.5 g/kg total lipid). Body weights of mice were monitored for 21 days. N=4 per group. Data is presented as mean ± SD.

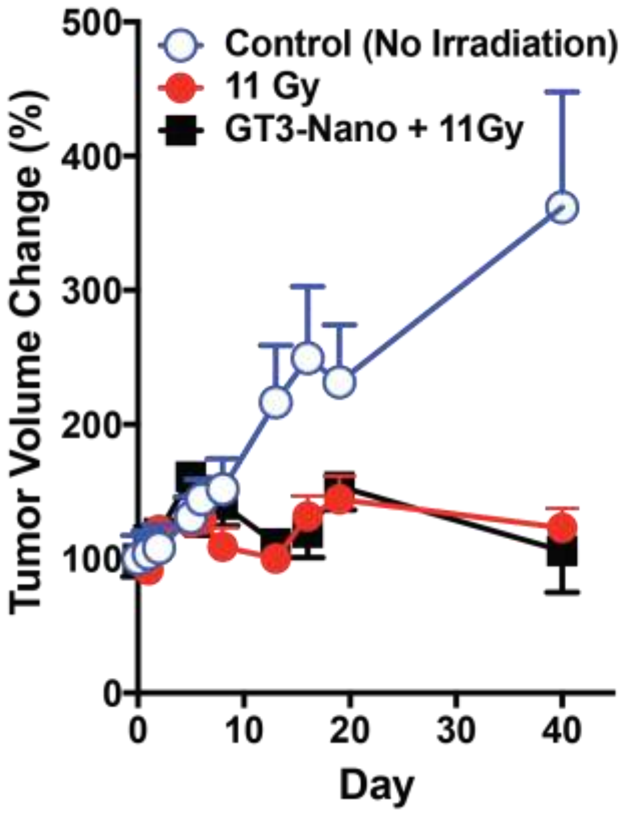


**Supplemental Figure 7. GT3 Effect on Tumor with focal irradiation.** 5 x $10^6$ MDA-MB-468-LUC cells in 100 uL Matrigel/PBS were injected s.c. to right flank of athymic nude mice. 5 mice per group. Tumor size became 50-100 $mm^3$ in two weeks and 10 mg/kg GT3 loaded liposomes were injected via i.v. 11 Gy was given to tumors using X-ray irradiator and tumor sizes were measured using caliper.

**Supplemental Table 1**. Biodistribution of GT3-loaded bone marrow-targeting liposomes labeled with [$^{3}$H]-GT3 and [$^{64}$Cu]-DOTA-Bn-DSPE. 5.5 MBq of $^{64}$Cu-labeled GT3-Nano and 370 kBq of $^{3}$H-labeled GT3-Nano were injected to mice *via* tail vein (n = 5 per group). Mice were sacrificed at the indicated time and major organs were collected and weighed. $^{64}$Cu counting was done as soon as the organs were collected and $^{3}$H counting was measured 5 days after the mice were sacrificed. The %ID/g was calculated by measuring weight and time-corrected measurement of radioactivity (n = 5 per group).

| | $^{3}$H | | $^{64}$Cu | |
|---|---|---|---|---|
| | 24 h | 48 h | 24 h | 48 h |
| Blood | 14.97 (8.85) | 7.54 (4.81) | 4.98 (0.25) | 0.50 (0.07) |
| Tumor (PC9) | N/A | N/A | 2.70 (0.36) | 0.74 (0.28) |
| Heart | 3.27 (0.47) | 2.98 (1.02) | 1.42 (0.12) | 0.62 (0.04) |
| Lungs | 3.07 (1.48) | 3.97 (1.45) | 1.72 (0.43) | 0.74 (0.13) |
| Liver | 6.53 (1.35) | 6.28 (0.63) | 14.40 (1.10) | 9.38 (0.92) |
| Spleen | 7.95 (1.61) | 8.26 (1.66) | 22.86 (8.14) | 19.20 (5.00) |
| Stomach | 2.09 (0.2) | 2.11 (0.45) | 0.20 (0.10) | 0.58 (0.41) |
| Sm. Intestine | 1.6 (0.3) | 1.66 (0.28) | 1.94 (0.54) | 1.74 (0.39) |
| Lg. Intestine | 1.87 (0.35) | 2.61 (0.66) | 0.60 (0.35) | 0.85 (0.41) |
| Kidney | 2.59 (0.46) | 2.41 (0.33) | 2.06 (0.51) | 0.72 (0.25) |
| Muscle | 1.53 (0.46) | 1.93 (0.39) | 0.14 (0.04) | 0.00 (0.00) |
| Marrow | 4.29 (3.22) | 7.44 (2.52) | 12.48 (2.68) | 6.98 (2.34) |

**Supplemental Table 2.** List of antibodies used for immunohistochemistry and flow cytometry.

| Antibody | Alternative names | Clone | Species | Company | Catalog Number |
|---|---|---|---|---|---|
| CD105 | Endoglin | poly | Rabbit | Abcam | ab107595 |
| CD31 | PECAM-1 | mono | Mouse | Abcam | ab24590 |
| Rb IgG | | poly | Goat | Abcam | ab150080 |
| Ms IgG | | poly | Goat | Abcam | ab150115 |
| CD3 | | mono | Hamster | eBioscience | 15-0031-83 |
| CD4 | | mono | Rat | eBioscience | 15-0041-83 |
| CD8 | | mono | Rat | eBioscience | 15-0081-83 |
| Gr1 | Ly6G/Ly6C | mono | Rat | eBioscience | 15-5931-82 |
| CD45R | B220 | mono | Rat | eBioscience | 15-0452-83 |
| CD19 | | mono | Rat | eBioscience | 15-0193-83 |
| Ter119 | CD253a | mono | Rabbit | eBioscience | 15-5921-82 |
| CD117 | c-Kit | mono | Rat | BioLegend | 105826 |
| Sca-1 | Ly6A/E | mono | Rat | BioLegend | 122520 |
| CD150 | SLAM | mono | Rat | BioLegend | 115910 |
| CD48 | BLAST-1 | mono | Hamster | BD Bioscience | 557485 |
| CD34 | RAM 34 | mono | Rat | eBioscience | 11-0341-85 |
| CD16/32 | Fcgr3/Fcgr2 | mono | Rat | BD Bioscience | 560829 |
